# Mosaic *hoxb4a* Neuronal Pleiotropism in Zebrafish Caudal Hindbrain

**DOI:** 10.1371/journal.pone.0005944

**Published:** 2009-06-17

**Authors:** Leung-Hang Ma, Beena Punnamoottil, Silke Rinkwitz, Robert Baker

**Affiliations:** 1 Department of Physiology and Neuroscience, New York University Medical Center, New York, New York, United States of America; 2 Brain & Mind Research Institute, University of Sydney, Camperdown, New South Wales, Australia; University of Maryland, United States of America

## Abstract

To better understand how individual genes and experience influence behavior, the role of a single homeotic unit, *hoxb4a*, was comprehensively analyzed *in vivo* by clonal and retrograde fluorescent labeling of caudal hindbrain neurons in a zebrafish enhancer-trap YFP line. A quantitative spatiotemporal neuronal atlas showed *hoxb4a* activity to be highly variable and mosaic in rhombomere 7–8 reticular, motoneuronal and precerebellar nuclei with expression decreasing differentially in all subgroups through juvenile stages. The extensive Hox mosaicism and widespread pleiotropism demonstrate that the same transcriptional protein plays a role in the development of circuits that drive behaviors from autonomic through motor function including cerebellar regulation. We propose that the continuous presence of *hoxb4a* positive neurons may provide a developmental plasticity for behavior-specific circuits to accommodate experience- and growth-related changes. Hence, the ubiquitous *hoxb4a* pleitropism and modularity likely offer an adaptable transcriptional element for circuit modification during both growth and evolution.

## Introduction

**Figure 1:**
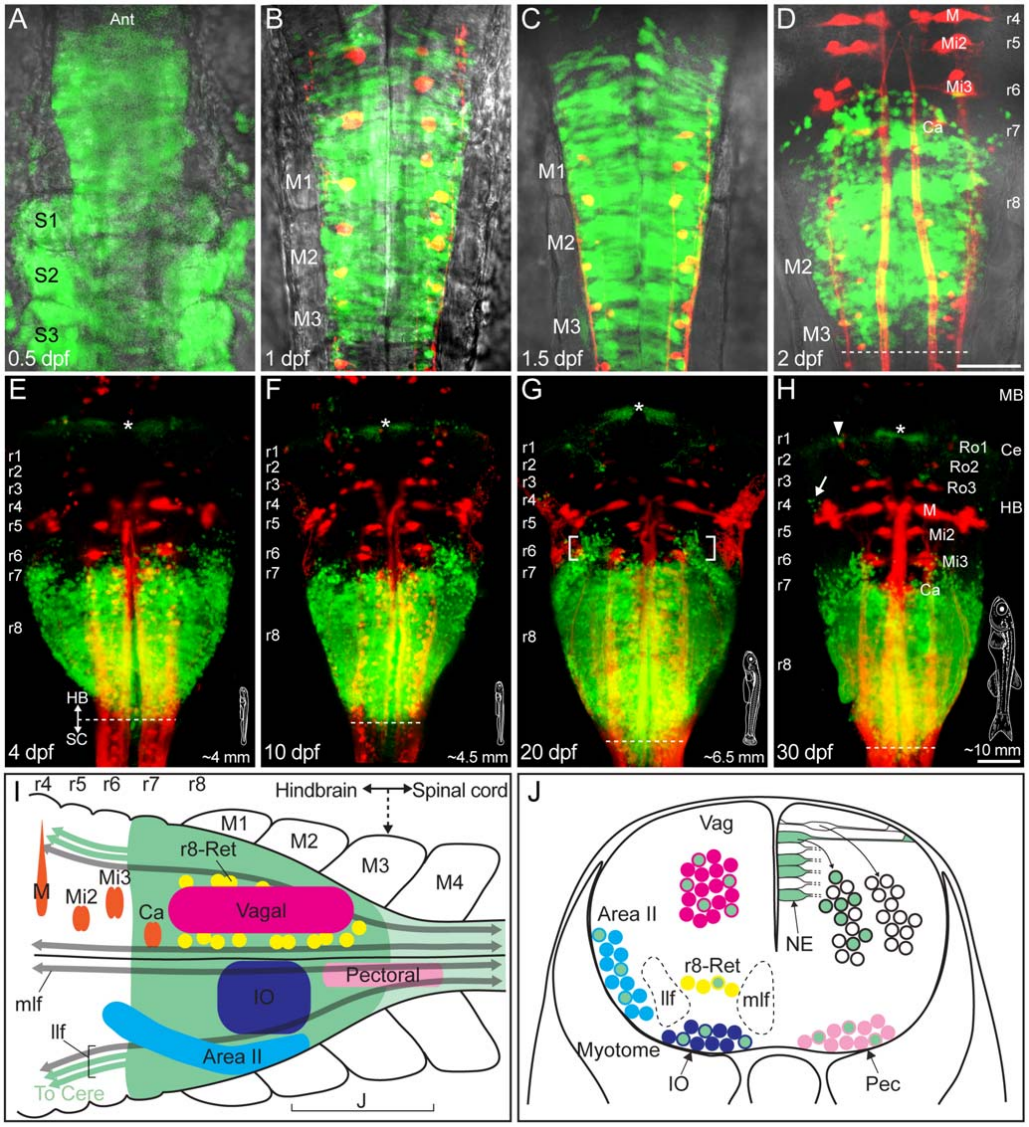
Overview of *hoxb4a* expression and hindbrain neuronal subgroups from 0.5 to 30 dpf. (A–H) Composite dorsal views of *hoxb4a*-YFP (green) and retrogradely labeled reticulospinal neurons (red) from confocal stacks of 133 µm (0.5 dpf), 116 µm (1 dpf), 116 µm (1.5 dpf), 176 µm (2 dpf), 148 µm (4 dpf), 172 µm (10 dpf), 190 µm (20 dpf) and 180 µm (30 dpf). Reticulospinal neurons in D, H are named according to Kimmel (1982) [32]. Bright-field images are overlaid in A–D. Arrow, bracket and asterisks in E–H mark *hoxb4a* activity observed in r4, r6 and cerebellum, respectively. Insets in E–H show the larvae at the corresponding stage with total body length indicated. (I–J) Schematics summarizing *hoxb4a* activity (green) with the location of r7–8 neuronal subgroups corresponding to rhombomeres and myotomes in dorsal (I) and coronal views (J). Abbreviations: Ant, anterior. Ce, cerebellum. HB, hindbrain. IO, inferior olive. M (in B–D, H), Mauthner cell. M (in I), myotome. MB, midbrain. mlf, medial longitudinal fasciculus. NE, neural epithelial cell. llf, lateral longitudinal fasciculus. r, rhombomere. Scale bars = 50 µm.

The hindbrain contains a broad neuronal diversity essential for survival in all vertebrates [1], [2]. Comparative developmental studies have shown it to be subdivided into segments, or rhombomeres, wherein serial repeats give rise to specific cranial motoneurons (IV–XII) along the anterior-posterior axis [3]. As the finite transition to spinal cord, the most caudal hindbrain rhombomeres (r7–8) are morphologically different from the rostral r2–6 by being more than twice as large and exhibiting no visible caudal boundary (Fig. 1A–J). In zebrafish, r7–8 gives rise to highly specialized neurons and circuits for cardiac-respiratory and intestinal function [4], locomotion [5] and posture [2], [6] along with the major precerebellar circuits responsible for motor coordination and learning [7], [8], [9]. Many r7–8 neurons such as the inferior olivary, vocal, electromotor and respiratory exhibit pacemaker-like rhythmic physiological properties suggesting that this compartment might be uniquely specified and evolutionary conserved for premotor circuitry underlying rhythmic behaviors [2], [10]. Ancestral conserved hindbrain genetic regulatory pathways also exhibit a combinatorial expression of Hox genes [11], [12]; however, the role of any 5′ Hox gene in either the formation or maturation, let alone the evolutional modification, of rhythmic circuits for any behavior remains unexplored [13].

Originally conceived as being important for body segmentation in insects, Hox genes have been conventionally suggested to pattern (i.e., segment) the embryonic vertebrate hindbrain during pre-rhombomeric neurulation stages [14]. Emerging evidence from loss-of-function experiments have shown Hox genes to act equally well at cellular levels, greatly influencing the neuronal diversity in the hindbrain [15], [16] and spinal cord [17], [18]. A number of Hox genes are expressed in the vertebrate hindbrain with different anterior boundaries, and Hox4 paralogs appear to be the major rhombomeric-specific group that delineates r7–8 [19]. The overlap of Hox4–6 genes in r7–8 suggests further genetic subdivisions; however, so far there is no evidence causally linking any given Hox gene, including the Hox4 paralogs, to either a specific hindbrain neuronal subtype or behavioral role. While Hox genes and their cofactors have been shown to be necessary links for creating certain spinal cord motoneuronal [17] and hindbrain somatosensory [20] pools, such roles have not been analyzed in defined pre-motor subgroups exhibiting the range of functions like those originating from r7–8.

Hox genes, like the majority of signaling proteins and transcription factors regulating development, operate independently in various tissues at different developmental stages. The phenomenon of one gene being responsible for more than one phenotypic characteristic has been termed ‘pleiotropism’ [21]. For example, roles have been suggested for Hoxb4 in oligodendrocyte maturation [22], self-renewal of hemopoietic stem cells [23], ventral body wall formation [24] and formation of vertebrate hindbrain neurons [25]. In a broader sense, mosaic pleiotropism has been suggested to be a major principle for the evolution of novel forms and structures [21]. Rhombomere specific Hox proteins have been shown to contribute to developmental plasticity during neuronal circuit formation and maturation with distant targets [20]. By alteration in *cis*-regulatory sequences, the spatiotemporal expression of a Hox protein can be reshaped and this epigenetic change could underlie a developmental plasticity in body form [26]. Although Hox genes exhibit a pleiotropism among macro-structures, it is not yet known how this attribute might be represented in different subgroups of neurons originating from a finite hindbrain compartment.

The objective of this study was to investigate the involvement of Hox genes in the development and maturation of individual identified hindbrain nuclei. To this end, the spatiotemporal activity of *hoxb4a* was documented from 1–30 days in specific subgroups of r7–8 neurons using high-resolution confocal microscopy in an enhancer-trap zebrafish line with yellow fluorescent protein (YFP) retroviral insertion [27], [28] that closely reproduces the endogenous mRNA expression.

The findings show *hoxb4a* mosaicism throughout all subpopulations of r7–8 neurons with minimal paralog presence in the reticulospinal system questioning a principal role in global patterning. The temporal profiles of *hoxb4a* activity differed between nuclei illustrating a neuronal pleiotropism and extending usage of this concept from the formation of macro-structures to a finite hindbrain compartment. As a result we propose that Hox genes have a role in developmental plasticity of hindbrain circuit wiring and maturation. We further suggest this could also provide a framework for inventing novel neuronal structure and/or function within the same species as well as in the evolution of derived behaviors such as vocalization [10], posture/locomotion [5], gaze [2] and cerebellar regulation [29].

## Results

### Spatiotemporal overview of *hoxb4a* activity in embryonic and juvenile development

Confocal microscopy was used to document *hoxb4a* activity reported in the CLGY 838 enhancer trap line [27]. Figure 1 provides a synopsis of global YFP expression from embryonic to juvenile stages (0.5–30 days) in the caudal hindbrain. *Hoxb4a*-YFP was first observed during the embryonic segmentation period (∼10–12 hrs; Fig. 1A). Caudal hindbrain expression continued into juvenile stages (Fig. 1B–H) while the size of the fish increased from <1 mm to ∼10 mm within 30 days (insets in Fig. 1E–H). Expression was still robust up to 60 days (data not shown).

While YFP was observed in both somites and hindbrain at ∼12 hrs (Fig. 1A), it was no longer detected in the somites by 1 day and became largely restricted to the caudal hindbrain through 30 days as r7–8 increased in length by ∼25% (or ∼35 µm; Fig. 1E–H). Use of the reticulospinal scaffold to identify rhombomeric borders showed that in addition to the primary r7–8 domain, *hoxb4a*-YFP was also expressed in cells dorsolaterally in r6 (bracketed in Fig. 1G). *Hoxb4a* cells were also observed laterally in r4 (arrows in Fig. 1H and Fig. S1C), as well as within the cerebellum (arrowheads in Fig. 1H and Fig. S1C). Activity was also maintained in the spinal cord (Fig. 1E–H and Fig. S1A–D; [25]), but at a relatively low level as compared to r7–8. The fluorescent intensity, and therefore the *hoxb4a* activity, in these transgenic fish were at similar levels throughout larval and juvenile development. Collectively these observations suggest a continuous role of *hoxb4a* beyond the initial neural induction and early hindbrain segmentation period.

A number of neuronal subgroups sent *hoxb4a*-YFP axons outside r7–8, but at distinctly different times. For example, axons of reticulospinal neurons reached the spinal cord by 1 day (Fig. 1B); vagal and pectoral motoneurons projected via the Xth and hindbrain occipital nerves, respectively, by 2 days (Fig. S1D, H); Precerebellar neurons, in particular those identified herein as Area II and the inferior olive, formed distinct clusters in the hindbrain by 4 days (Fig. S1A–D, F–H). Area II neurons target granule cells in the caudal lobe of the cerebellum [29] and reach the contralateral external granule cell layer through Larsell's commissure (Fig. S1F). Inferior olivary neurons directly contact all Purkinje cells and in the case presented here, are likely projecting to the ganglionic layer of the corpus cerebelli (Fig. S1F; [30]). Axonal projections to the midbrain and diencephalon also were observed by 4 days but could not be correlated with distinct nuclei (Fig. S1G). The major neuronal subgroups in the caudal hindbrain that could be unequivocally identified by retrograde labeling with fluorescent dyes are summarized in Fig. 1I–J. Confocal *in vivo* imaging in this enhancer trap line was used to document three-dimensional heterogeneity of *hoxb4a* activity within the caudal hindbrain reticular, precerebellar and motoneuronal subgroups throughout embryonic to juvenile stages.

### Mosaic *hoxb4a* expression in hindbrain r7–8

**Figure 2:**
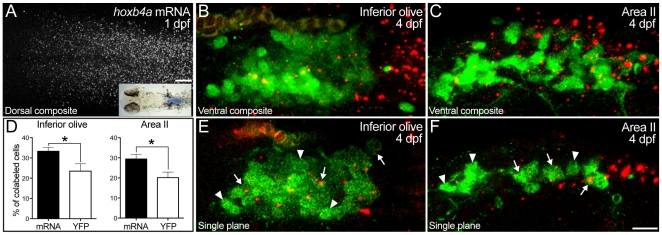
Mosaic *hoxb4a* expression within hindbrain nuclei. (A) *Hoxb4a* expression in hindbrain r7–8 of a 1 dpf larva detected by fluorescent and colormetric procedures (inset). (B–C) Ventral composites of the precerebellar inferior olive (B) and Area II (C) nuclei (20 µm confocal stacks) retrogradely labeled from the cerebellum at 4 dpf (green), with *hoxb4a* mRNA (red) detected by fluorescent *in situ* hybridization. (D) Graphs showing only a subset of inferior olive and Area II neurons expressed *hoxb4a* mRNA (solid bar) and *hoxb4a*-YFP (open bar). P-values from Student t-test were 0.04 for both the inferior olive and Area II. (E–F) Single plane images showing mosaic *hoxb4a* expression in the olive (E) and Area II (F). Arrows and arrowheads point to cells that did and did not expression *hoxb4a*. Scale bar = 20 µm (A) and 10 µm (B–C and E–F).

**Figure 3:**
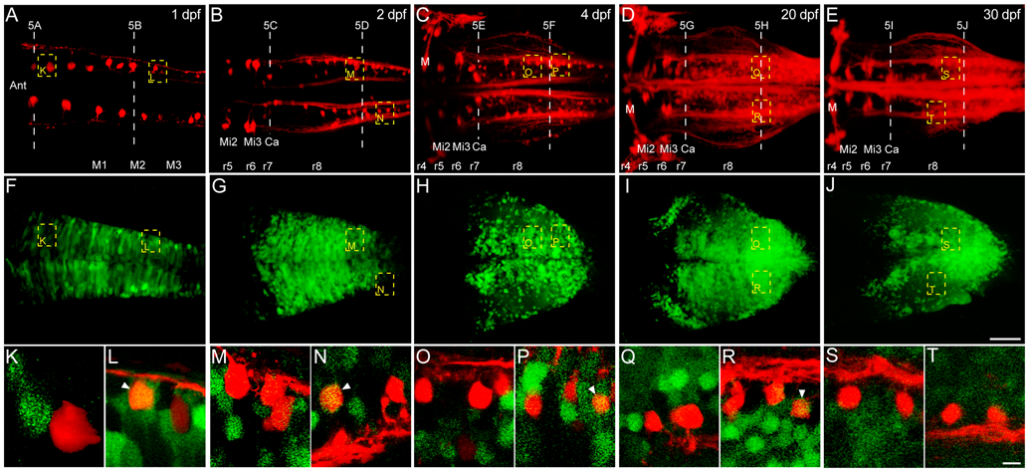
*Hoxb4a* activity in reticulospinal neurons. (A–J) Composite dorsal views showing retrogradely labeled reticulospinal neurons (A–E) and corresponding *hoxb4a*-YFP expression (F–J) from 116 µm (1 dpf), 176 µm (2 dpf), 148 µm (4 dpf), 190 µm (20 dpf) and 180 µm (30 dpf) confocal stacks. (K–T) High magnification single plane images showing T-interneurons and *hoxb4a* expressing cells in r8. Arrowheads point to co-labeled cells. Abbreviations: Ant, anterior. Ca, Caudal Rhombencephalon. M, Mauthner cell. M1–3, myotome pair 1–3. Mi2, Middle Rhombencephalon level 2. Mi3, Middle Rhombencephalon level 3. Scale bars = 50 µm (A–J) and 5 µm (K–T).

Globally, the *hoxb4a*-YFP enhancer trap line recapitulated the endogenous expression in r7–8 (Fig. 1). However, YFP expression was mosaic with in various hindbrain circuits (below) raising the question of whether the endogenous mRNA expression also exhibits mosaicism, and in particular, within identified neuronal subgroups. To address this issue, fluorescent *in situ* hybridization of *hoxb4a* mRNA (Fig. 2A) was performed in conjunction with retrograde labeling from the cerebellum. At a cellular level, *hoxb4a* expression was unexpectedly mosaic within r7–8 (Fig. 2A and data not shown) like that observed in the enhancer trap line (Fig. 1 and 3–7). Within the identified precerebellar nuclei, endogenous mRNA expression was found to be mosaic in both inferior olive and Area II neurons (Fig. 2B–C and E–F; Movie S1). Surprisingly, only a subset of inferior olivary (33.33±1.90%) and Area II neurons (29.45±2.33%; Fig. 2D) expressed *hoxb4a*. The corresponding observations using the enhancer trap line were 20.16±2.74% and 23.49±3.65% (Fig. 2D), respectively. Therefore, the enhancer trap line reports ∼70% (p<0.05) of the endogenous *hoxb4a* expressing neurons in both circuits (Table S1). The YFP reporter thus represents nearly all of the endogenous mRNA expressing neurons showing the enhancer trap line to be a viable tool for studying the mosaic and spatiotemporal aspects of *hoxb4a* activity *in vivo*.

Similar to mRNA expression, a mosaic *hoxb4a*-YFP pattern was observed within r7–8 throughout 1–30 days (Fig. S2A–E) in the enhancer trap line. Nuclear counterstain with Hoechst 34580 showed that *hoxb4a* cells appeared as clusters (for example, Fig. S2C) that most likely represented clones as strongly supported by the analysis of single cells labeled during early gastrulation at 6–8 hrs (below). *Hoxb4a* cells were concentrated in a ∼100 µm dorsal domain within r7–8 (below). The percentage of YFP expressing cells sampled in 20–40 µm regions from the dorsal surface were estimated from 1–30 dpf. The numbers obtained thus represent the highest possible percentage of *hoxb4a* cells within r7–8. From 1–10 days, the percentage of YFP expressing cells increased from ∼35% to ∼45% and then decreased to ∼25% by 30 days (Fig. S2E; Table S2). The spatiotemporal profiles of *hoxb4a* activity did not support an all-inclusive presence in caudal hindbrain r7–8 cells at any time during development.

### *Hoxb4a* activity in neurons sending axons outside r7–8

Retrograde labeling at sites marked in Fig. S1 identified reticular and precerebellar neurons along with pectoral and vagal motoneurons (also see Methods and Materials). *Hoxb4a* activity was assessed in these r7–8 neuronal subgroups at sequential times to investigate the extent of heterogeneity in different behavioral circuits (Fig. 3–7). In short, *hoxb4a*-YFP expressing cells were found in all the identifiable subgroups except for the r7-reticular neurons, with the percentage decreasing during hindbrain expansion and maturation. These data are summarized in Fig. 7K, Fig. S3 and Table S4.

**Figure 4 pone-0005944-g004:**
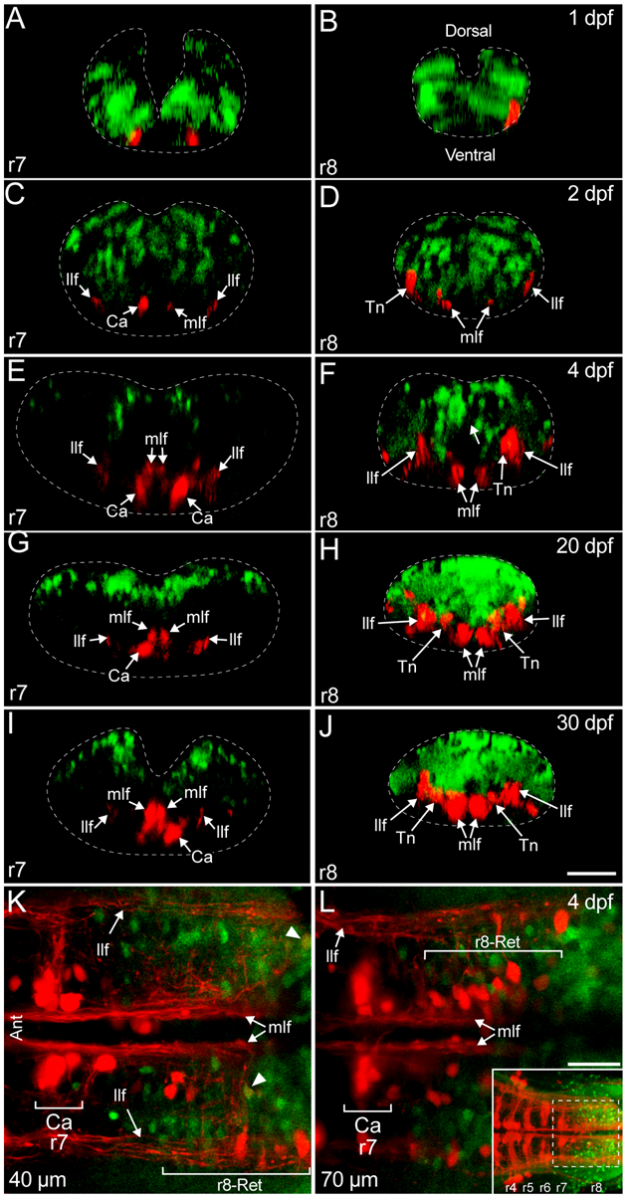
*Hoxb4a* activity along the dorsoventral axis in r7–8. (A–J) Optically reconstructed transverse sections at the level of r7 (A, C, E, G, I) and r8 (B, D, F, H, J) in 1, 2, 4, 20 and 30 dpf transgenic zebrafish. Dashed lines outline the hindbrain boundaries in the sections with anterior-posterior position indicated in Fig. 4 (A–E). (K–L) Single plane images acquired from the ventral side of an isolated brain at 40 µm (K) and 70 µm (L) depth showing *hoxb4a*-YFP expression and reticulospinal neurons from r7 to rostral r8 at 4 dpf. Arrowheads point to r8-reticular neurons that expressed *hoxb4a*-YFP. Inset in L is an overview of the caudal reticular scaffold and *hoxb4a* cells imaged from the ventral side with the box indicating the viewing field in K–L. Abbreviations: Ant, anterior. Ca, Ca neuron. Llf, lateral-longitudinal fasciculus. Mlf, medial-longitudinal fasciculus. r8-Ret, r8-reticular neurons. Scale bars = 50 µm (A–J) and 10 µm (K–L).

**Figure 5 pone-0005944-g005:**
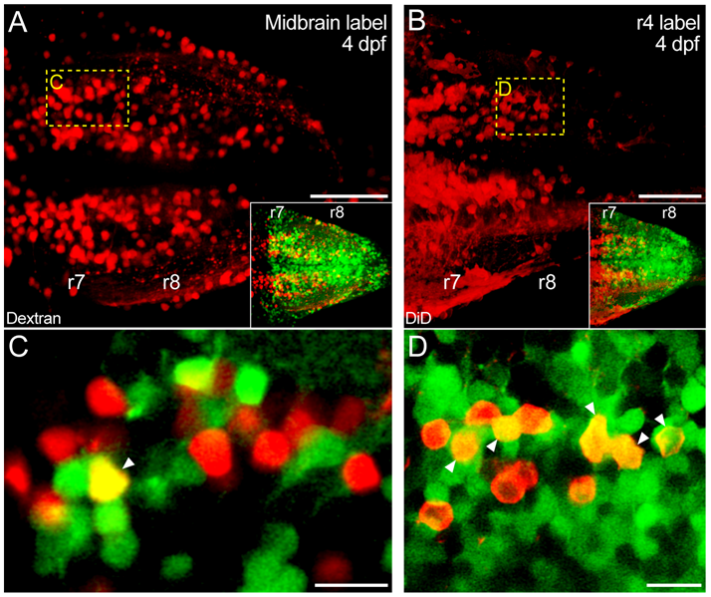
*Hoxb4a* activity in rostrally projecting neurons. (A–B) Composite dorsal views from 110 µm (A) and 125 µm (B) confocal stacks showing neurons retrogradely labeled from the midbrain (A) and r4 (B) at 4 dpf. Insets show the corresponding *hoxb4a*-YFP expression. (C–D) Single plane high magnification images showing *hoxb4a*-YFP expression and retrogradely labeled neurons. Arrowheads point to co-labeled cells. Anterior is to the left. Scale bars = 50 µm (A–B) and 10 µm (C–D).

**Figure 6 pone-0005944-g006:**
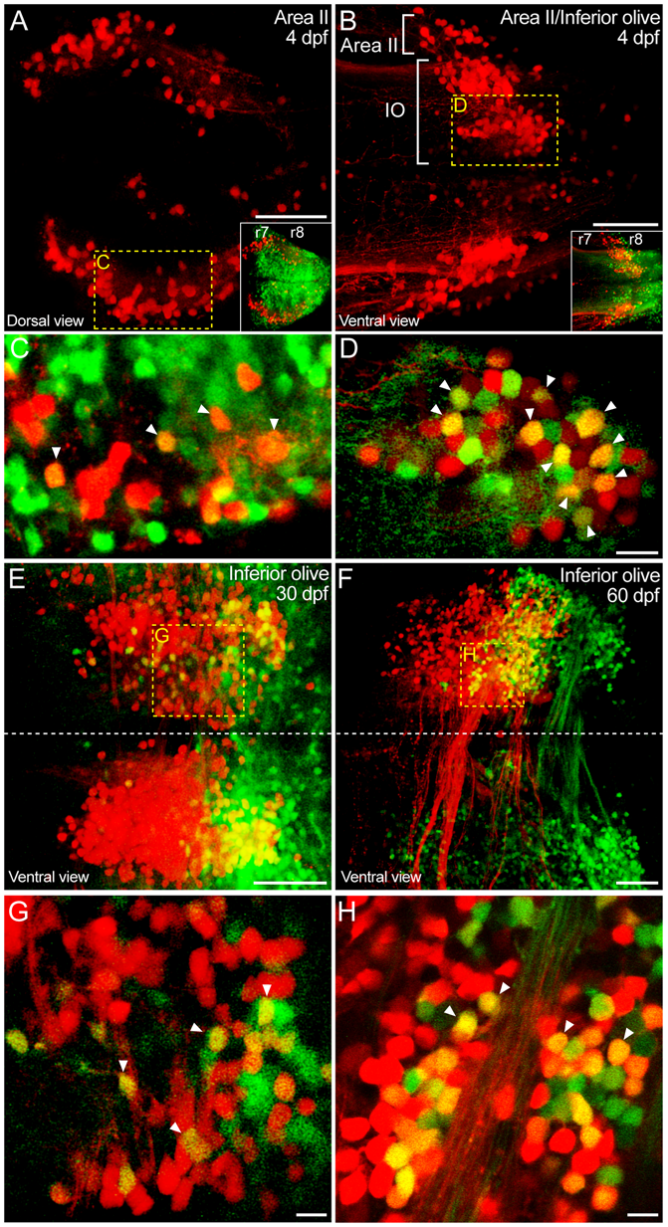
*Hoxb4a* activity in precerebellar neurons. (A–B, E–F) Composite dorsal views from 110 µm (A), 125 µm (B), 60 µm (E) and 75 µm (F) confocal stacks showing neurons retrogradely labeled from the cerebellum at 4 dpf (A–B) as well as the inferior olive at 30 dpf (E) and 60 dpf (F). Insets in A–B show the corresponding *hoxb4a*-YFP expression at 4 dpf. (C–D, G–H) Single plane high magnification images showing *hoxb4a*-YFP expression and retrogradely labeled neurons. Arrowheads point to co-labeled cells. Anterior is to the left. Scale bars = 50 µm (A–B, E–F) and 10 µm (C–D, G–H).

**Figure 7:**
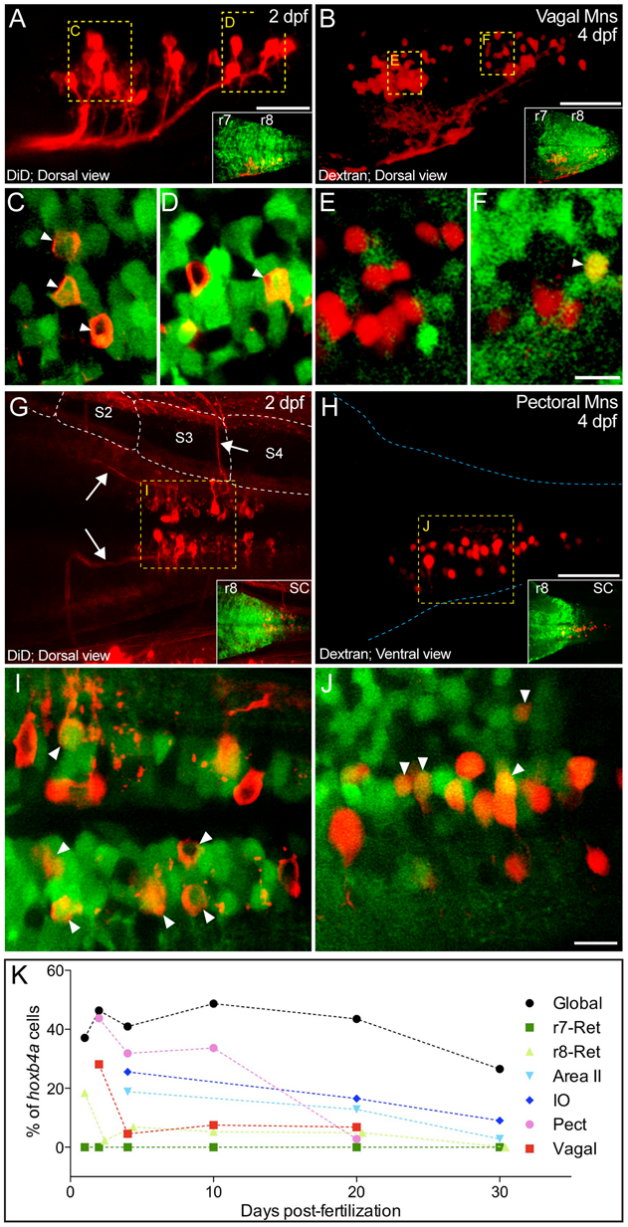
*Hoxb4a* activity in vagal and r8-pectoral motoneurons. (A–B, G–H) Composite dorsal views from 125 µm (A), 180 µm (B), 130 µm (G) and 70 µm (H) confocal stacks showing retrogradely labeled vagal (A–B) and pectoral (G–H) motoneurons at 2 and 4 dpf. Insets show the corresponding *hoxb4a* expression. (C–F, I–J) Single plane high magnification images showing *hoxb4a*-YFP expression and retrogradely labeled motoneurons. Arrows point to co-labeled cells. Anterior is to the left. Scale bars = 50 µm (A–B, G–H) and 10 µm (C–F, I–J). (K) Percentage of *hoxb4a* cells in each identified neuronal subgroup versus time from 2–30 dpf.

#### 1) Reticular neurons with axons descending to the spinal cord

Retrograde labeling from rostral spinal cord identified the classically defined hindbrain reticular scaffold shown in Figs. 1 and 4 [31]. Neurons within the *hoxb4a* activity domain, i.e., predominantly r7–8, were labeled at 1 day (Fig. 3A, F). By 2 dpf, a more extensive reticular scaffold was present and previously named neurons such as the Mauthner cell, Mi2, Mi3 and Ca groups, could be identified morphologically and served as landmarks to distinguish individual rhombomeres (Fig. 3B) [32]. Neurons were continuously added to the reticular scaffold from 4 to 30 dpf (Fig. 3C–E; Table S4) while the primary *hoxb4a* activity domain remained confined to r7–8 (Fig. 3G–J).

Reticular neurons in r7 located in the caudal rhombencephon and originally called the Ca group [32] (Fig. 3B) aligned with the anterior boundary of YFP expression (Fig. 3G). In all larvae examined, and at different stages (Summarized in Fig. 7K; Fig. S3; Table S4), YFP was never observed in any r7-reticular neuron, even at 1 dpf when the most immature neuronal morphology was observed as illustrated by the two most rostral groups within the expression domain (Fig. 3K and 4A). Optically reconstructed transverse sections of r7 from 2 dpf onwards showed *hoxb4a* cells to occupy a ∼100 µm dorsal domain and be physically separated from the reticular scaffold through 30 dpf (Fig. 4A, C, E, G, and I). The entire r1–7 reticular scaffold of neurons was located ventrally, and did not exhibit any *hoxb4a* activity (Fig. 4C) even though the number of neurons increased considerably throughout 30 dpf (Fig. 4E, G, I). Observations from the ventral side in isolated hindbrains provided a higher resolution for the reticular scaffold and confirmed that *hoxb4a*-YFP was not expressed in r7-reticular neurons at 4 dpf after more neurons were added (Fig. 4K–L; Movie S2). These findings suggest that *hoxb4a* is not involved cell autonomously in either the early establishment or subsequent maintenance of the reticulospinal system.

Reticular neurons in r8 were identified at 1 dpf (Fig. 3A and F) and, in contrast to those in r7, ∼18% expressed YFP (Fig. 3L and 7K; Table S4). From 2–30 dpf, considerably more r8-reticular neurons were retrogradely labeled (Fig. 3B–E) and, by in large, they resembled the previously described T-reticular interneurons [33] (Fig. 3M–R). The percentage of *hoxb4a* cells within the population decreased to ∼2% by 2 dpf and remained <7% through 20 dpf (summarized in Fig. 7K and Table S4). Surprisingly, at 30 dpf (and also at 60 dpf), none of the r8-reticular neurons expressed YFP (Fig. 3S–T and 7K; Table S4). Unlike the spatial separation observed in r7, many *hoxb4a* neurons were in a close apposition to labeled r8-reticular neurons from 1–30 dpf (Fig. 4B, D, F, H and J). Imaging the hindbrain from the ventral side convincingly showed r8-reticular neurons intermingled with YFP expressing cells (Fig. 4K–L; Movie S2). In contrast with r7, the YFP activity in r8 reticular neurons indicates that *hoxb4a* could play a role in the early, but not late, circuit development.

#### 2) Reticular neurons with axons ascending towards the midbrain and rostral hindbrain

Midbrain labeling revealed two large medial populations of neurons in r7–8 at 4 dpf (Fig. 5A). These ventrally located neurons only partially overlapped with the *hoxb4a* dorsal domain and ∼9% expressed YFP (Fig. 5C; Table S4). In contrast, discrete dye application directed at the center of rhombomere 4 labeled neurons in r7–8 with axons projecting locally or rostrally to targets in the midbrain and diencephalon (Fig. S1G). The majority of identified neurons were located mediodorsally, completely overlapped with the *hoxb4a* dorsal domain, yet only ∼32% expressed YFP at 4 dpf (Fig. 5B and D). These percentages demonstrated that neurons with connectivity within and/or between the hindbrain rhombomeres exhibited a much higher *hoxb4a* activity than those ascending to either the midbrain or descending to the spinal cord (Table S4).

#### 3) Precerebellar neurons

Retrograde labeling of the entire cerebellar plate at 4 dpf revealed two distinct subgroups in r7–8 largely representing, based on adult anatomy [29], Area II and inferior olivary neurons (Fig. 6A–B; Movie S3) that provide the classical mossy and climbing fiber input, respectively, to the cerebellum as shown in Fig. S1F. Dorsolateral columns of neurons (herein called Area II) were identified bilaterally across r6–8 overlapping with the dorsal *hoxb4a* domain in r7–8, but notably separate from the YFP cells in r6 (Fig. 6A and inset). While the column appeared continuous, YFP was only found in labeled r7–8 neurons as opposed to those located in r6 (inset in Fig. 6A). At 4 dpf, ∼19% of the r7–8 Area II neurons expressed YFP (arrowheads in Fig. 6C) and there was no discernable distribution pattern (i.e., random) along the anterior-posterior axis (Fig. 6C). The percentage of *hoxb4a* neurons decreased to 13% and 3% at 20 and 30 dpf (summarized in Fig. 7K and Table S4) while retaining a random distribution throughout the column.

The labeled inferior olivary neurons were located ventrolaterally in mid-r8 (Fig. 6B and inset) and they exhibited ∼26% YFP expression at 4 dpf (arrowheads in Fig. 6D). Similar to that described for Area II, YFP expressing neurons were distributed randomly within the inferior olive from 4–20 dpf. The percentages of *hoxb4a* inferior olivary neurons decreased to 16% and 19% at 20 and 30 dpf (Fig. 7K; Table S4). However, interestingly, *hoxb4a* activity was found to be largely concentrated in the caudal one third of the olive at 30 (Fig. 6E) and 60 (Fig. 6F) dpf but the distribution remained mosaic (Fig. 6G–H). Such an activity profile in the inferior olive suggests a different requirement of *hoxb4a* transcriptional activity among subdivisions of the precerebellar circuits during growth and maturation (see Discussion).

From 4–20 dpf the total number of neurons increased in Area II by ∼2 fold and in the inferior olive by ∼4 fold while the number showing *hoxb4a* activity increased by ∼1.5 and ∼2 fold, respectively (Table S4). These numerical data demonstrate that both *hoxb4a* positive and negative neurons were recruited to precerebellar circuits. This comparison also shows a similar trend of *hoxb4a* activity in both precerebellar circuits as summarized in Fig. 7K.

#### 4) Vagal and pectoral motoneurons

Vagal motoneurons were identified dorsomedially in r8 within the *hoxb4a* activity domain (Fig. 7A–B and insets). YFP expression was observed in ∼28% of neurons at 2 dpf (Fig. 7C–D), but then dropped to ∼5% by 4 dpf (Fig. 7E–F; Table S4) during a ∼4 fold increase in neuron number. During a further ∼2 fold expansion of the nucleus, *hoxb4a* activity was maintained at a similar level throughout 10 and 20 dpf (∼7%; Fig. 7K; Table S4).

Pectoral motoneurons were located at the level of myotome 3 and 4 along the anterior-posterior axis at 2 dpf (Fig. 7G). The motor column spanned the hindbrain/spinal cord boundary as could be seen from the YFP expression pattern (inset in Fig. 7G). Motoneurons were situated ventromedially, partially contained within the hindbrain *hoxb4a* domain (inset in Fig. 7G). Axons exited the hindbrain at successive anterior-posterior levels and coalesced into distinct axon bundles that directly innervated the pectoral fin bud (arrows in Fig. 7G). From 2 to 4 dpf, the pectoral motoneurons pool size increased by <10% (Table S4) while maintaining a columnar location across the hindbrain/spinal cord boundary (Fig. 7H and inset). YFP expression was observed in ∼44% of the pectoral motoneurons at 2 dpf (Fig. 7I), and unlike the drastic decrease observed in vagal motoneurons, 30–35% remained YFP positive at 4–10 dpf (Fig. 7J–K). While the r8 pectoral motoneuron pool expanded considerably by 20 dpf (∼3 fold in number), *hoxb4a* activity dropped to <3% and only a few cells expressed YFP (Fig. 7K; Table S4).

Since both YFP positive and negative neurons were added to the vagal and pectoral motoneuron pool from 2 to 20 dpf (Table S4), recruitment to either nucleus was not *hoxb4a* dependent. Vagal motoneurons are part of the autonomic nervous system that innervates numerous branchial-derived structures like the heart/viscera whereas pectoral motoneurons innervate a diversity of fin muscles that are somato-motor-related. Nonetheless, distribution of the *hoxb4a* neurons within the two nuclei remained random at all stages examined (Movie S4–5). These data did, however, demonstrate *hoxb4a* activity was differentially regulated in these two pools of r8 motoneurons and could be correlated to a difference in circuit maturity (see Discussion).

Collectively, the temporal and spatial profiles of reticular, precerebellar and motoneurons provided an anatomical map of different neuronal subgroups in the caudal hindbrain together with their *hoxb4a* activity pattern (Fig. 1I–J, 7K, Fig. S3 and Table S4). Except for r7-reticular neurons, *hoxb4a* was mosaically present in all hindbrain r7–8 neuronal subgroups. More significantly, activity was differentially regulated both between and within different neuronal populations. Overall a minority, <40%, of the neurons in a given subgroup exhibited a peak *hoxb4a* activity that occurred within the first 4 dpf of development. Even though both Hox positive and negative neurons were added during the larva to juvenile transition, the expression percentage decreased at a subgroup-specific rate (Fig. 7K; Fig. S3).

### Clonal analysis in *hoxb4a* background after single-cell injection

**Figure 8:**
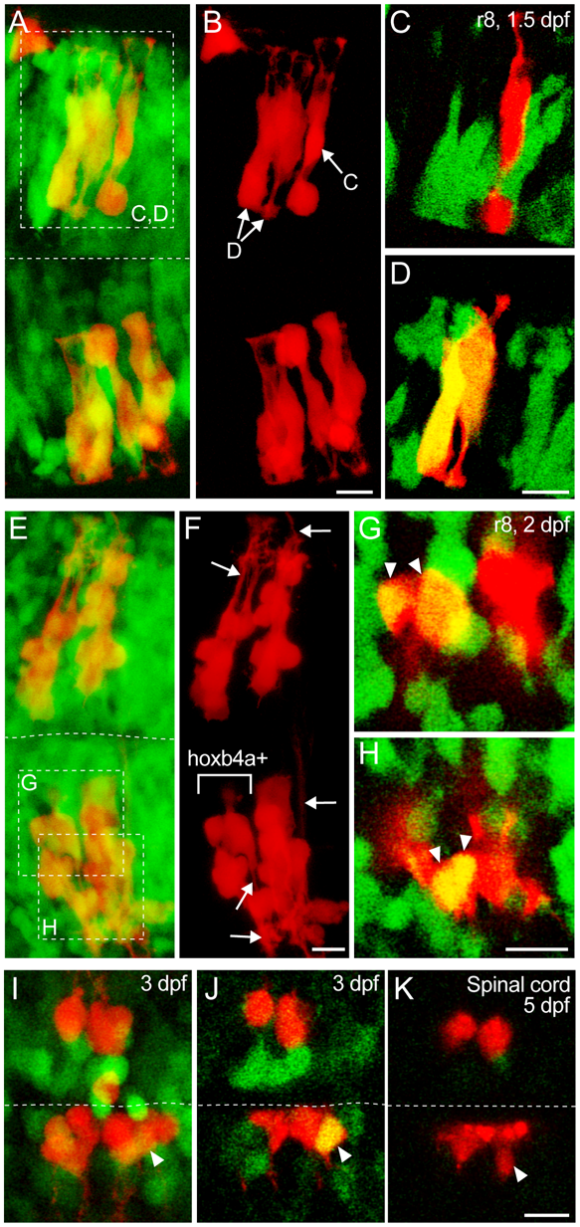
Clonal analysis by single cell injection. (A–B, E–F, I) Composite dorsal views from 165 µm (A–B), 170 µm (E–F) and 130 µm (I) confocal stacks showing progenitors from a single cell injected during the gastrula period (red) and *hoxb4a*-YFP (green). (C–D, G–H, J–K) Single plane high magnification images showing the progenitor cells and *hoxb4a*-YFP expression. Arrowheads point to the co-labeled cells. Arrows in F mark the processes (dendrite/axon) extending from the neurons at 2 dpf. Anterior is to the left. Dashed lines marked the midline. Scale bars = 10 µm.

In order to determine whether the observed mosaicism and randomness of *hoxb4a* activity was linked to a clonal neuronal origin, single-cell injections were implemented during the gastrula period. Individual clones were followed and studied in the hindbrain and the spinal cord. Five key questions were addressed and are illustrated by the three representative cases presented in Fig. 8 (A–D; G–H; I–K) along with a summary in Table S3: Is *hoxb4a* expression 1) cell-autonomous, 2) temporally confined, 3) clonal, 4) down regulated and 5) necessary for neuronal differentiation and/or survival?

The first example (Fig. 8A–D) shows a single cell injected during early gastrula stage at ∼6–8 hrs that expanded into bilateral clones in r8 at 1.5 dpf (Fig. 8A–B). All the labeled cells at this time were neuroepithelial (Fig. 8B). While the bottom clone in Fig. 8A–B was completely YFP negative, the top clone exhibited mosaic *hoxb4a* activity. This demonstrated an asymmetry between clones across the midline (see Discussion). Single optical sections at high magnification (Fig. 8C–D, marked in Fig. 8A–B) showed neuroepithelial cells in the top clone shared the same origin and remained in close proximity within r8. Yet they could be either YFP negative (Fig. 8C) or positive (Fig. 8D). The data suggested *hoxb4a* expression could be initiated non-cell autonomously and constrained after cells divide across the midline (see Discussion).

The second example (Fig. 8E–H) shows an injected cell that expanded into four bilateral clones in r8 by 2 dpf (Fig. 8E–F). Again, asymmetry was observed between clones across the midline as neuroepithelial cells in only one of the four clones expressed YFP at 1 dpf. Expression was maintained in the neurons at 2 dpf (marked in Fig. 8E–F) as shown at a higher magnification (Fig. 8G–H). Mosaicism was not observed within the positive clone at 2 dpf suggesting *hoxb4a* activity to be clonal with heterogeneity originating at the neuroepithelial cell stage. The labeled cells at 2 dpf became neurons with dendritic arborizations and axons (arrows in Fig. 8F). Neuronal maturation occurred independent of either the presence or absence of *hoxb4a* activity and this paralog is therefore not essential for either neuronal differentiation or survival for every neuron in r7–8.

The third example (Fig. 8I–K) shows an injected cell that split into two bilateral clones in the rostral spinal cord, with asymmetry across the midline and mosaicism observed in the bottom clone. One ventrolaterally located neuron in the clone expressed *hoxb4a*-YFP at 3 dpf as shown in single optical section in Fig. 8J (arrowhead). At 5 dpf, the same cell, as judged by following its location within the clone, no longer expressed YFP (arrowhead in Fig. 8K). These data are consistent with a down regulation of *hoxb4a* activity during circuit maturation.

Collectively, the clonal analyses showed mosaic *hoxb4a* activity to originate at the neuroepithelial stage and to be confined to neuronal progenies. By contrast, while not required for either neuronal survival or differentiation for every neuron in r7–8, *hoxb4a* activity was nearly always present in a subset of clones in which expression could be down regulated during neuronal maturation.

## Discussion

### *Hoxb4a* mosaicism and pleiotropism

The majority of the genes regulating development, including Hox, function independently in different cell types at different stages so the same protein is utilized in the formation of structurally different germ layers and body parts. This broad genetic property, termed pleiotropism, has been described in invertebrate models [21]. The current study extends this usage to individual nuclei originating from a finite segmental compartment in the vertebrate hindbrain.

Instead of exhibiting a 100% presence in all subgroups, *hoxb4a* activity was observed mosaically in a minority of the reticular, precerebellar and motoneurons (Fig. 4–7; Table S2 and S4). Activity was subgroup-specific and independently regulated within each nucleus. Pectoral and vagal motoneuron pools illustrated this feature best because *hoxb4a* activity decreased at a different rate between 2–20 dpf (Fig. 7). The temporal activity profiles did not correlate with global activity in r7–8 that was maintained at ∼40%. *Hoxb4a* mosaic neuronal pleiotropism was a continuous feature during the developmental acquisition of diverse behaviors [2] with the presence of activity beyond embryonic stages suggesting a role in circuit plasticity and maturation.

### Evaluating *hoxb4a* activity in the developing hindbrain

Global YFP expression in this reporter line shares the enhancer module of the endogenous gene and resembles the pattern of *hoxb4a* mRNA within the r7–8/spinal cord (Fig. 1–2; [25]). Temporally, the onset of YFP expression in the mesoderm (∼10 hpf) and neural keel (∼11 hpf) matched that detected by *in situ* hybridization [11]. At the cellular level, endogenous *hoxb4a* expression was mosaic, as demonstrated by fluorescent *in situ* hybridization in the two precerebellar nuclei (Fig. 2; Table S1; Movie S1). The enhancer trap line was shown to report ∼70% of the endogenous expression in two representative hindbrain nuclei (Fig. 2D). The slightly lower endogenous gene activity indicates the YFP reporter did not fully capture the whole range of the gene expression pattern within r7–8. Since the enhancer trap line reported gene activity at a cellular level during dynamic *in vivo* imaging, it provided greater insight to the spatiotemporal characterization of *hoxb4a* mosaicism and allowed a better evaluation of neuronal pleiotropism in the zebrafish hindbrain.

### Possible roles for *hoxb4a* in neural development

Conventionally, Hox genes have been suggested to pattern the neural tube along the anterior-posterior axis and determine segmental identity in the vertebrate hindbrain [14]. Such a model would suggest a ubiquitous Hox activity in all types of neuron within the expression domain. In contrast, the current data showed that *hoxb4a* activity is mosaic in rhombomere 7–8 as well as in individual neuronal circuits throughout 30 dpf (Fig. 1, 7 and Table S4). Such mosaicism would suggest *hoxb4a*, and perhaps other Hox genes as well, do not act globally, but instead exert their influence at a cellular level throughout vertebrate neural development.

In our clonal analysis experiments, asymmetry of *hoxb4a* activity was frequently observed between related clones on opposite sides of the midline (Fig. 8A–H). This asymmetry suggested that clonal restriction of *hoxb4a* activity follows the mirror-symmetric division of neuroepithelial cells across the midline between 14–18 hpf [34]. Such a time course demonstrated that *hoxb4a* expression onset was not cell-autonomous as neuroepithelial cells with the same clonal origin could respond to extrinsic signaling like observed for retinoic acid [35] and FGF [36] gradients. Neuroepithelial cells without *hoxb4a* activity could differentiate into neurons with dendrites and axons (Fig. 8E–H). Such observation indicates that a single Hox4 paralog like *hoxb4a* may not be a general transcription factor for neuronal differentiation. In contrast to the conventional model, a Hox paralog may not be sufficient to give rise to all the neuronal diversity in rhombomeres 7–8.

In all circuits studied (except the r7 reticular neurons), *hoxb4a* activity was sustained during later development in a subset of neurons. Such a continuous presence would then suggest a role for *hoxb4a* during neuronal growth, late differentiation and/or refinements that might contribute to the specification of discrete neuronal phenotypes within a circuit. Many of the hindbrain nuclei are heterogeneous, for instance, the inferior olive, pectoral and vagal motor nuclei contain neurons with different morphology, physiology and projection targets [4], [6], [29]. The mosaic *hoxb4a* activity could be part of a transcriptional system for such subdivision. Interestingly, clusters of YFP cells were never observed in any of the labeled nuclei (Fig. 3–7) even though *hoxb4a* activity was shown to be clonal (Fig. 8E–H), suggesting that cells were not recruited to a given circuit based on either their clonal origin or *hoxb4a* activity. The above properties may not be unique to *hoxb4a* as the other Hox paralogs might behave similarly during neuronal patterning.

Globally, *hoxb4a* was present in less than half of all cells in the caudal hindbrain (Fig. S2E; Table S2) and the percentage was far less in any of the nuclei studied (e.g. reticular and motoneuronal) throughout the first 30 dpf (Fig. 3–7; Table S4). A matter of considerable interest would be whether the other Hox4 paralogs fill the gap such that all r7–8 neurons might be Hox4 positive. Observations of mRNA expression and YFP reporter from other Hox4 enhancer trap lines ([25]; unpublished data) showed *hoxa4a* and *hoxd4a* expression to be mosaic, like the case for *hoxb4a*, suggesting the activity of Hox4 paralogs might not be completely overlapping. This conjecture is strongly supported by the notably transient *hoxa4a* activity (i.e., not observable after ∼10 dpf) as compared to the *hoxb4a* activity that was still very robust at 60 dpf. Therefore, Hox4 paralogs might only partially overlap, both spatially and temporally, in some r7–8 neurons. Collectively, r7–8 quite likely contains a huge repertoire of neurons with various Hox4 paralog combinations and/or gene doses, which perhaps may have been a selected for consequence of the Hox cluster duplication [37].

Hox regulatory networks in the spinal cord are suggested to determine motoneuronal phenotypes and establish correct peripheral muscle innervation [18], [38]. By contrast, our observations of hindbrain vagal and pectoral motoneurons suggest a single Hox4 paralog would be insufficient to specify the phenotypes for an entire motor nucleus. Our data of *hoxb4a* mosaicism is more in agreement with data from *hoxb2^−/−^* mice where the r4–5 facial motor nucleus exhibited maturational defects, including path-finding and termination, instead of being absent [39]. Such alleged ‘maturational roles’ for Hox genes might only be elucidated by physiological assessment of neural circuit performance through behavioral quantification [9] since any genetic manipulation leading to possible maturational defects in either membrane properties or neurophysiological signaling would not be easily discernable at the level of a morphological marker.

With only a couple of exceptions (e.g. r4 and midbrain label; Fig. 5A–D) all the neurons evaluated in this study gave rise to long-range excitatory connections (e.g., precerebellar and motoneuronal). Hox activity in an equally large population of intra/inter-rhombomeric inhibitory pathways was not taken into account. Excitatory (glutamatergic) and inhibitory (glycinergic and GABAergic) neurons have been shown to organize in clusters along the dorsoventral axis in the zebrafish hindbrain [40]. Such a distinctive organization was not seen with *hoxb4a* activity (Fig. 4). The ubiquitous mosaicism throughout the dorsoventral axis and intra-/inter-rhombomeric axonal projections [25] suggests that some *hoxb4a* neurons are inhibitory. If so, then gene activity does not likely confer a specific neurotransmitter phenotype. A mosaic *hoxb4a* activity in both excitatory and inhibitory neuronal circuits would therefore define another level of pleiotropism.

### *Hoxb4a* activity correlates with neuronal circuit maturity

The presence of *hoxb4a* activity observed through juvenile stages strongly suggests a role in post-embryonic development (Fig. 1). During this period, existing neurons expand their axonal and dendritic structures while new neurons are continually added to the blueprint in order to accommodate changes in the sensory and motor periphery during acquisition of adult body form. However, rather than being maintained at initial levels, *hoxb4a* activity exhibited a decrease in all identified subgroups from 2–30 dpf (Fig. 4–7; Fig. S3 and Table S4). Such an inverse temporal profile in all neuronal subgroups suggests neuronal maturity and *hoxb4a* activity may be temporally correlated as illustrated in pectoral and precerebellar circuits.

In zebrafish, pectoral fins exhibit a progressive but considerable change in musculature, central innervations pattern [6] and fin movement [41] between larval and juvenile stages. The temporal *hoxb4a* profile matches fairly well with this developmental timeline as activity was maintained at a relatively high level (30–40%) in the pectoral motoneurons from 1–10 dpf (Fig. 7G–J), during which time period the number of fin muscles increased from 2 to 6 accompanied by change in innervation pattern [6], [41]. The *hoxb4a* activity only decreased later to <3% at 20 dpf (Fig. 7K; Table S4). Perhaps the *hoxb4a* activity from 1–10 dpf provides necessary transcriptional plasticity [42] for modifications of existing circuits which, in turn, accommodates the increasing complexity in fin structure, innervation and movement control. By 20 dpf, fin morphology reaches an adult conformation requiring less tuning of the central neural circuits. If so, then *hoxb4a* activity might be down regulated in the more mature neurons like in those observed in the single-cell injection experiments (Fig. 8 I–K).

Precerebellar circuits further illustrate the suggestion that Hox genes provide a developmental plasticity during neuronal circuit maturation. Like described for pectoral fins, the cerebellum of nearly all teleosts first appears as a plate in the early larval stages (Fig. S1) and then exhibits considerable growth during development. Subsequently, three distinct subdivisions (corpus, valvula and caudal lobe) are formed that receive a multimodal input of auditory, visual, proprioceptive and lateral line signals [29]. Of particular interest here were the neurons, derived from r7–8 that provide the afferent precerebellar input to the caudal lobe shown to be essential for oculomotor learning and memory [7]. This population appears as a homogeneous subgroup called Area II that, functionally, exhibits a continuous range of physiological properties signaling eye and head velocity [8]. *Hoxb4a* activity was maintained at 10–20% in this population from 4–20 dpf and decreased to <5% by 30 dpf with no obvious distribution pattern within the nucleus. Continuous presence of *hoxb4a* within the nucleus might be envisioned to provide a transcriptional plasticity for fine-tuning optokinetic and vestibuloocular circuitry as improved performance and adaptation are proportional to zebrafish size [9].

Based on an innervation pattern deduced from the adult teleost cerebellum [43], the inferior olive is subdivided into rostral, medial and caudal parts that innervate the caudal lobe, corpus and valvula, respectively (unpublished observations; [29]). Like described for precerebellar Area II, *hoxb4a* activity was present at 15–25% in all 3 olivary subdivisions from 4–20 dpf with a random distribution (Fig. 6F–H). This observation suggests that *hoxb4a* might contribute to the early developmental wiring of hindbrain neurons with both the granular (Area II) and ganglionic (inferior olive) layers of the cerebellum.

At 30 and 60 dpf, however, *hoxb4a* activity was found mainly in the caudal part of the olive that projects to the valvula (Fig. 6E–H), which suggests a unique transcriptional requirement for this particular precerebellar circuit. The lateral line provides the major input to the valvula and its sensory field continues to expand with fish size. As a result, motion detecting neuronal circuits in the caudal part of the inferior olive are subjected to continuous enlargement. Persistent and focused presence of *hoxb4a* activity in this particular subdivision of the inferior olive would provide a transcriptional plasticity accommodating expansion and growth of the lateral line system which, in turn, underlies consolidation in this precerebellar circuit [8].

In contrast, the rostral and medial subdivision of the olive projecting to the caudal lobe and corpus exhibit minimal *hoxb4a* activity at 30 and 60 dpf (Fig. 6E–H). In this case, both the auditory and visual surrounds are largely invariant due to the early maturation of the eye and ear sensory elements during juvenile stages, hence reflecting a decreased requirement for transcriptional competence. Collectively, the activity profiles in the pectoral (locomotion) and oculomotor (motion sensing) precerebellar circuits strongly implicate a role for *hoxb4a* in circuit formation that accommodates growth-related reorganization in sensory and motor fields accompanying the changes in body form.

Recently, one of the homeobox proteins, OTX2, has been shown to be transported through a neuronal network in the visual cortex [44]. Homeobox proteins can be secreted and internalized through an endocytosis-independent, non-vesicular mechanism [45] that includes *hoxb4* [46]. By such a proposed mechanism, *hoxb4a* could couple changes in hindbrain r7–8 with those in an innervated target, like the cerebellum, during circuit maturation.

### Absence of *hoxb4a* in the embryonic reticulospinal scaffold

Reticular neurons in r7 are born between 12–27 hpf [47] but none were *hoxb4a* positive at 1 dpf (∼24–28 hpf; Fig. 3–4). Transient expression in the early r7 precursors of reticular neurons is unlikely because *hoxb4a* activity would have to be rapidly down regulated by the time the cell bodies were labeled from axons in the rostral spinal cord (Fig. 3–4). Consistent with this surmise, *hoxb4a* was not observed in neurons subsequently added to the r7-reticular group by 4 dpf (Fig. 4K–L and Movie S2). Observations from two other Hox4 enhancer trap lines (*hoxa4a* and *hoxd4a*) along with mRNA expression patterns (Fig. 1B–D in Punnamoottil 2008) suggest that r7-reticular neurons might never exhibit Hox 4 activity. Therefore, perhaps no Hox4 paralogs, particularly *hoxb4a*, may be required cell-autonomously for r7-reticular neurons to achieve their proper innervation and functional operation. The reticulospinal scaffold forms the most precocious neuronal circuits in the zebrafish hindbrain [47] that function very early for escape behavior [5]. If, as suggested above, *hoxb4a* acts to provide a developmental plasticity for juvenile circuit growth and expansion, then the requirement may be less for reticulospinal circuits.

Hox genes have been envisioned to segment the hindbrain into rhombomeres (e.g. Hox4 for r7–8), however, it has been argued that Hox2 paralogs (*a* and *b*) are not required for generating r3–5 [39]. Our data agree with the latter observation as the mosaic onset of *hoxb4a* activity would be insufficient to pattern an entire rhombomeric segment and, thus, it alone could not be a causal factor for segmentation. The concentration of YFP cells in a ∼100 µm dorsal domain in r7 from 4 dpf onwards suggests a possible role of *hoxb4a* in hindbrain dorsoventral patterning like for other Hox paralogs [39]. Comparative studies have concluded that co-linear Hox expression [48] and hindbrain segmentation are independent processes in evolution as well as in development [49] that have become more coordinated as evolution progressed [48], [50]. The mosaic *hoxb4a* activity observed in r7–8 is consistent with this idea and implies that Hox genes may play a more important role in neuronal patterning than segmentation.

### Evolution in closely related hindbrain nuclei

Overall, this study has demonstrated a mosaic *hoxb4a* pleiotropism to be present during neuronal maturation from 1–30 dpf. Based on the different spatiotemporal dynamics among caudal hindbrain nuclei, we propose Hox genes could act as a facilitator in neuronal subgroup evolution. *Cis*-regulatory sequences very often control spatiotemporal expression of a gene [26] and have been suggested to play an important role during the evolution of form [21]. Mutation in *cis*-regulatory sequences would modify the expression profiles of Hox genes without affecting protein function [21]. Temporal changes in Hox expression have been demonstrated to alter neuronal projections [20]. Notably, an alternation in Hox expression was shown to consequently allow for the emergence of novel, functional neuronal circuits [51]. Mutations built up in the *cis*-regulatory sequences during evolution might modify the mosaic pattern, which could lead to the acquisition of new neuronal Hox code and, thus, the creation of novel circuits from the existing neuronal prototypes. Partial redundancy in paralog function would further provide an extraordinary biological flexibility for the occurrence of both developmental and evolutionary events. This view is supported by the observation that a majority of caudal hindbrain nuclei exhibit similar pacemaker-like physiological properties. These circuits uniquely originated in r7–8 and are responsible for many rhythmic behaviors. It would not be surprising if the mosaic *hoxb4a* pleiotropism is part of the underlying genetic mechanisms that gave rise to more derived premotor circuitry using the more ancient evolutionary blueprints.

### Pleiotropy and modularity of Hox genes

The concept of modularity has emerged in developmental, evolutionary and molecular biology to address a network of interactions, like subgroup specializations occurring in r7–8, that can be subdivided into relatively autonomous highly connected components [52]. Since the evolution of higher organisms apparently do not suffer from a ‘cost of complexity’ by mutations that grossly affect a few traits, the Hox gene pleiotropy would appear to be a remarkable means to minimize effects on multiple phenotypic characters [52]. The impact for evolution in the hindbrain is immediately clear, as pleiotropy appears to have been introduced into nearly every nuclear module that in turn is subjected to the processes of natural selection.

Behavioral phenotypes result from complex interactions between nature and experience. Development provides the stage for this production, but the detail of how genes and experience interact within the developing hindbrain to create complex phenotypes is unknown. The long standing ‘problem with anatomy’ is to explain how evolvability and adaptive radiation occur within a finite region, like a hindbrain compartment. That is, for example, to account for how pectoral, vocal, electromotor and other evolutionarily-derived central neurons and circuits originated from r7–8 in teleost fishes let alone any further extrapolation to other, more derived, neuronal circuits in vertebrates.

The mosaic Hox gene pleiotropy described in this paper suggests a plausible rationale and practical origin for modularity, in which selection pressures can favor developmental processes that reinforce a beneficial bias in existing neuronal variations [53]. An emerging theme in developmental biology is to understand the effects of a single gene (i.e. like *hoxb4a*) on a much larger molecular network. This may be a plausible experimental approach to identify the genuine origin of modularity, hence the genotype–phenotype relationship. Observing behaviors emerge during development in the zebrafish and establishing a correlation with Hox gene expression is a sound first step; however to tackle any of the comparative innovations mentioned, such as vocalization, will require considerable neurogenetic approaches in other teleosts.

In conclusion, based on these data, we believe that the overall developmental contribution of Hox genes has been altogether underestimated by only considering the conventional roles suggested in embryonic hindbrain segmentation and patterning [14]. Rather, we propose that the maintained expression of Hox transcription factors contributes to a progressive improvement of behavior-specific circuit performance as new neurons are continuously integrated in the context of experience-related requirements [9]. Experimentally, our observations fit well with the literature published on the concepts of neuronal “pleiotropism” [21] and “modularity” [53] as currently discussed in developmental and evolutionary biology. We suggest such neuronal pleiotropism may be an economic usage of Hox genes in the engineering and modification of various hindbrain neuronal circuits during maturation. This additional genetic trait would also offer a robust modifiable transcriptional substrate for neuronal circuits to evolve [21].

## Materials and Methods

### Zebrafish husbandry

Zebrafish were maintained according to standard procedures [54] and used in accordance with the *Guide for the Care and Use of Laboratory Animals* (1996) following protocols approved by the NYU School of Medicine Institutional Animal Care and Use Committee.

### Retrograde labeling

Zebrafish were anesthetized with 0.02% ethyl 3-aminobenzoate methanesulfonate (MS 222, Sigma) and immobilized in 2% low gelling agarose (type VII, Sigma) prepared in 30% Danieau's solution. After dye application, larvae were placed in modified artificial cerebrospinal fluid (ACSF; 67 mM NaCl, 2.9 mM KCl, 10 mM HEPES, 2.1 mM CaCl_2_, 1.2 mM MgCl_2_, 10 mM glucose, 164 mM sucrose, pH 7.5, 323.8 mOsm) for at least 3 hours for dye diffusion and/or transportation before preparation for imaging (see Confocal Microscopy below).

#### 1) Reticulospinal neurons labelling

The spinal cord was lesioned at the fifth myotome level with a sharp tungsten needle transecting the cord and overlaying muscles dorsal to the notochord. A crystal of Alexa Fluor 647 dextran (10,000 MW, anionic, Invitrogen) was placed at the lesion site for 2–3 minutes.

#### 2) Midbrain, cerebellum and r4 labeling

Skin covering the brain was removed by sharp tungsten needles and fine forceps under ACSF. For midbrain labeling, optic tectum was removed to expose the underlying midbrain. Alexa Fluor 488 or 647 dextran (3% in 0.2 KCl with 0.1% triton X-100) was pressure injected into the midbrain or cerebellum at 10–20 psi using glass electrodes of 5–10 µm tip. Injections were monitored under a dissection microscope and repeated until the dye filled up the space in the midbrain and cerebellum where projections were observed from the caudal hindbrain (Fig. S1F–G). For r4 labeling, DiD (Invitrogen) coated glass electrode tips were inserted into r4 area using the otic vesicle as a landmark.

#### 3) Vagal motoneuronal labelling

For larvae at 2 dpf, vagal motoneurons were labeled by inserting a DiD coated glass electrode tip into the vagal nerve located caudal to the otic vesicle [55]. For 4 and 20 dpf larvae, a lesion was made with a sharp tungsten needle between the otic vesicle and cleithrum after which a crystal of Alexa Fluor 647 dextran was placed at the site.

#### 4) Pectoral motoneuronal labelling

For larvae at 2 dpf, pectoral motoneurons were labeled by inserting DiD coated glass electrode tips into the fin buds. For 4 and 20 dpf larvae, Alexa Fluor 647 dextran (3% in 0.2 KCl with 0.1% triton X-100) was pressure injected into the abductor and adductor muscles of the fins at 10–20 psi using glass electrodes of 5–10 µm tip and repeated until both muscles were filled with the dye.

### Single cell injection

Embryos at early gastrula period (∼6–8 hrs) were dechorionated manually, immobilized with 3% methylcellulose (in 30% Danieau's solution) and oriented using the dorsal embryonic shield as landmark. Electrodes with 2–3 µm tips filled with 3% Alexa Fluor 647 dextran solution (in 0.2M KCl) and exhibiting resistance of 100–200 MΩ at 1 nA DC current were used to inject single deep layer cells (DEL) by a 10 nA DC current. Cells giving rise to hindbrain and rostral spinal cord were targeted at locations between 20–40° longitude and 60–90° latitude [56]. Dye loading was monitored visually using a florescent microscope. Embryos were removed from the methylcellulose after injection and incubated in 30% Danieau's solution until the desired stages for imaging.

### Hoechst staining

Skin covering the brain in 1 to 30 dpf zebrafish was removed under ACSF using sharp tungsten needles and fine forceps. Exposed brains were incubated with Hoechst 34580 (Invitrogen; 5 µg/ml in ACSF, 0.1% DMSO) for 1 hour before preparation for imaging (see Confocal Microscopy below).

### *In situ* hybridization

*Hoxb4a* was cloned using the following primer sequences: 5′-TATAGAATTCATGGCCATGAGTTCCTATTTG-3′ and 5′-TATAGCGGCCGCGCTTGCTCGGCTCTGATT-3′. A DIG labeled RNA probe against *hoxb4a* was made using T3 and T7 polymerase (Promega). Retrograde labeling from the cerebellum was performed in 4 dpf larvae as described above. Brains were isolated and fixed with 4% paraformaldehyde at room temperature for 4 hours in order to preserve perinuclear localization of mRNA. *In situ* hybridization was performed using standard protocol [57], except the hybridization temperature was set to 68°C. Signals were either detected colormetrically by alkaline phosphatase conjugated anti-DIG antibody (Roche) and BCIP/NBT (Roche), or fluorescently by peroxidase conjugated anti-DIG antibody (Roche) and Alexa Fluor 647 tyramide (as part of the TSA kit#16; Invitrogen) according to manufacturer's instruction. After signal development, retrogradely labeled precerebellar neurons were detected using both anti-Alexa Fluor 488 and Alexa Fluor 488 conjugated anti-rabbit antibodies (Invitrogen).

### Confocal imaging

Confocal imaging was performed with a Zeiss META 510 confocal system using Achroplan 10×/0.25, Achroplan 40×/0.8 W, Achroplan 40×/0.65, Achroplan 63×/0.9 W and Achroplan 63×/0.8 objectives. The excitation wavelengths used were 405 nm (Hoechst), 488 nm (Alexa Fluor 488), 514 nm (YFP) and 633 nm (DiD, Alexa Fluor 647) and to avoid bleed-through, channels were scanned sequentially.

Zebrafish larvae were anesthetized with 0.02% MS 222 and immobilized in 2% low gelling agarose for imaging. For experiments that involved 1–2 dpf zebrafish, embryos were raised in 0.003% 1-phenyl-2-thiourea (PTU, Sigma) in 30% Danieau's solution. For experiments that used larvae 4 dpf or beyond, the skin covering the brain was removed using sharpened tungsten needles (under modified ACSF containing 0.02% MS 222) to expose the brain for imaging from the dorsal side. For imaging from the ventral side, brains were acutely isolated after labeling and mounted between bridged coverslips. Post-acquisition image processing was performed using NIH ImageJ, Adobe Photoshop and Illustrator. Rhombomeric identity outside r7–8 was inferred by referring to the classes of reticulospinal neurons retrogradely labeled from the spinal cord [58].

In every experiment, 6–8 different embryos were examined to ensure reliability of observations. *Hoxb4a* activity in any identified group of neurons was determined by summing data from 3–4 specimens (Fig. 7K) exhibiting a similar degree of labeling and then acquiring an average of the percentages calculated from individual experiments (Fig. S3). The numbers (N) and statistical analyses of experiments are listed on Table S1–4.

## Supporting Information

Figure S1 Live imaging and immunohistochemically detected hoxb4a activity in the midbrain, cerebellum, hindbrain and spinal cord. Composite dorsal (A) and side (C) views of hoxb4a expression in a live 5 dpf transgenic zebrafish from 210 µm and 150 µm confocal stacks, respectively. (B, D) Dorsal (B) and side (D) views of hoxb4a-YFP using immunohistochemistry (anti-YFP) in a fixed 6 dpf fish. Horizontal (E, G) and coronal sections (F, H) with section planes indicated in (B, D). Target sites for retrograde labeling are marked by 1 (spinal cord), 2 (Xth nerve), 3 (pectoral fin), 4 (cerebellum), 5 (midbrain) and 6 (r4). Abbreviations: AII, Area II; Ce, cerebellum; D, diencephalon; HB, hindbrain; llf, lateral longitudinal fascicle; IO, inferior olive; MB, midbrain; mlf, medial longitudinal fascicle; Oc N, occipital nerve; OV, otic vesicle; Pon N, pontine nucleus; SC, spinal cord; Vagal N, vagal nerve. B, D and E–H are cropped high magnification illustrations of Figs. 5K, 5J, 5C, 5T, 5D and 4B, respectively, (from [25]).
(6.92 MB TIF)

Figure S2 Mosaic hoxb4a activity. (A–D) Single plane images showing hoxb4a (green) and Hoechst nuclear counterstain (red) acquired from the dorsal 60 µm of r7–8 at 2 (A), 4 (B), 10 (C) and 20 (D) dpf in transgenic zebrafish. Dashed lines mark the ventricular surface. (E) Graph showing the percentage change of hoxb4a cells from 1 to 30 dpf in dorsal r7–8. Scale bars = 10 µm.
(2.98 MB TIF)

Figure S3 Percentage of hoxb4a cells in each identified neuronal subgroup versus time from 2–30 dpf. Percentages are presented as mean±S.E.M. calculated from individual experiments (also see Table S2)
(0.58 MB TIF)

Table S1 Statistical analysis of hoxb4a activity in precerebellar nuclei using mRNA expression and the hoxb4a-YFP reporter. Means are expressed as mean±S.E.M.
(0.13 MB TIF)

Table S2 Statistical analysis of nuclear counter-stain experiments. Means are expressed as mean±S.E.M.
(0.14 MB TIF)

Table S3 Summary of single-cell injection experiments.
(0.13 MB TIF)

Table S4 Statistical analysis of neuronal subgroup labeling experiments.Numbers of labeled neurons and percentages are expressed as mean±S.E.M. Reticulospinal neurons were not morphologically distinguishable at 1 dpf. The two rostral-most groups of labeled neurons within the hoxb4a-YFP domain were considered to be progenitors that eventually give rise to r7-reticular neurons.
(0.38 MB TIF)

Movie S1 Mosaic hoxb4a expression in the inferior olive at 4 dpf. Stack of confocal sections imaged at 63× from the ventral side of an isolated brain processed with in situ hybridization showing hoxb4a mRNA (red) and inferior olivary neurons (green) retrogradely labeled from the cerebellum.
(3.76 MB MOV)

Movie S2 Reticulospinal scaffold labeling in 4dpf hoxb4a-YFP zebrafish. Stacks of confocal sections imaged at 40× and 63× from the ventral side of an acutely isolated brain showing hoxb4a (green) and reticulospinal neurons (red) retrogradely labeled from the spinal cord. Rhombomeres 3–8 were identified by the presence of characteristic reticulospinal neurons. The imaged stack acquired at a higher magnification shows r7-reticular neurons did not exhibit any hoxb4a activity while a small percentage of r8-reticular neurons expressed YFP mosaically.
(5.04 MB MOV)

Movie S3 Precerebellar neurons in 4 dpf hoxb4a-YFP zebrafish. Stacks of confocal sections imaged at 40× and 63× from the ventral side of an acutely isolated brain show hoxb4a (green) along with inferior olivary and Area II neurons (red) retrogradely labeled from the cerebellum. Inferior olivary and Area II neurons were located ventromedially and laterally, respectively. Imaged stack acquired at a higher magnification illustrates mosaic hoxb4a activity within both precerebellar nuclei.
(4.44 MB MOV)

Movie S4 Vagal motoneurons in 4 dpf hoxb4a-YFP zebrafish. Stacks of confocal sections imaged at 40× and 63× from the dorsal side of an intact zebrafish brain showing hoxb4a (green) and vagal motoneurons (red) retrogradely labeled via the Xth nerve. Vagal motoneurons were located medially in r8. The imaged stack acquired at a higher magnification illustrates mosaic hoxb4a activity within the nucleus.
(4.44 MB MOV)

Movie S5 Pectoral motoneurons in 2 dpf hoxb4a-YFP zebrafish. Stacks of confocal sections imaged at 40× and 63× from the dorsal side of an intact zebrafish brain showing hoxb4a (green) and pectoral motoneurons (red) retrogradely labeled from the fin bud. Pectoral motoneurons located ventromedially at the level of somite 3–4. The imaged stack acquired at a higher magnification shows mosaic hoxb4a activity within the nucleus.
(5.04 MB MOV)
