# Supplementary figures and images for: Mosaic hoxb4a Neuronal Pleiotropism in Zebrafish Caudal Hindbrain
Source: PLoS One. 2009 Jun 17;4(6):e5944. doi: 10.1371/journal.pone.0005944 (PMC2693931; doi:10.1371/journal.pone.0005944)

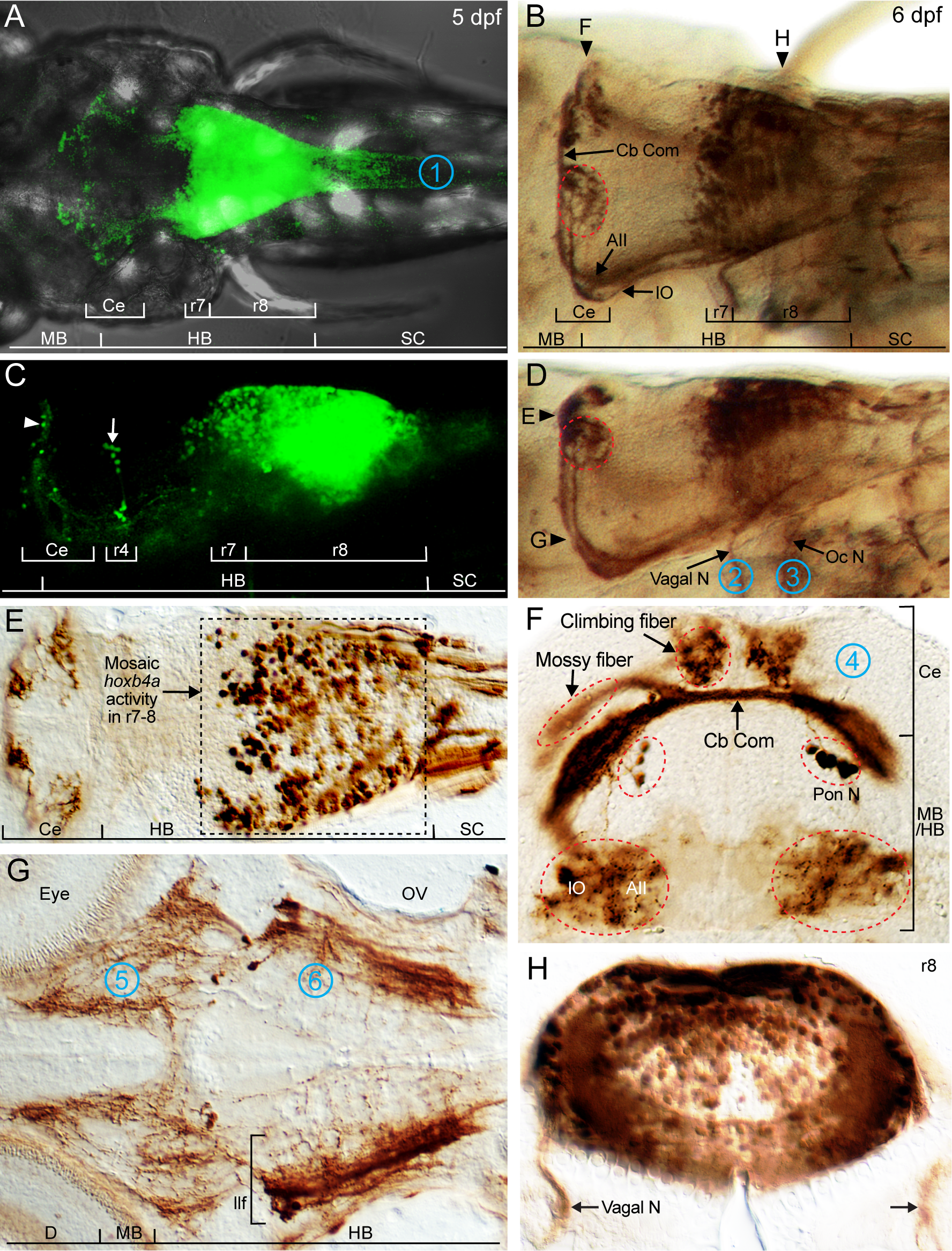

Supplement: Figure S1 — Live imaging and immunohistochemically detected hoxb4a activity in the midbrain, cerebellum, hindbrain and spinal cord. Composite dorsal (A) and side (C) views of hoxb4a expression in a live 5 dpf transgenic zebrafish from 210 µm and 150 µm confocal stacks, respectively. (B, D) Dorsal (B) and side (D) views of hoxb4a-YFP using immunohistochemistry (anti-YFP) in a fixed 6 dpf fish. Horizontal (E, G) and coronal sections (F, H) with section planes indicated in (B, D). Target sites for retrograde labeling are marked by 1 (spinal cord), 2 (Xth nerve), 3 (pectoral fin), 4 (cerebellum), 5 (midbrain) and 6 (r4). Abbreviations: AII, Area II; Ce, cerebellum; D, diencephalon; HB, hindbrain; llf, lateral longitudinal fascicle; IO, inferior olive; MB, midbrain; mlf, medial longitudinal fascicle; Oc N, occipital nerve; OV, otic vesicle; Pon N, pontine nucleus; SC, spinal cord; Vagal N, vagal nerve. B, D and E–H are cropped high magnification illustrations of Figs. 5K, 5J, 5C, 5T, 5D and 4B, respectively, (from [25]). (6.92 MB TIF) [file pone.0005944.s001.tif]

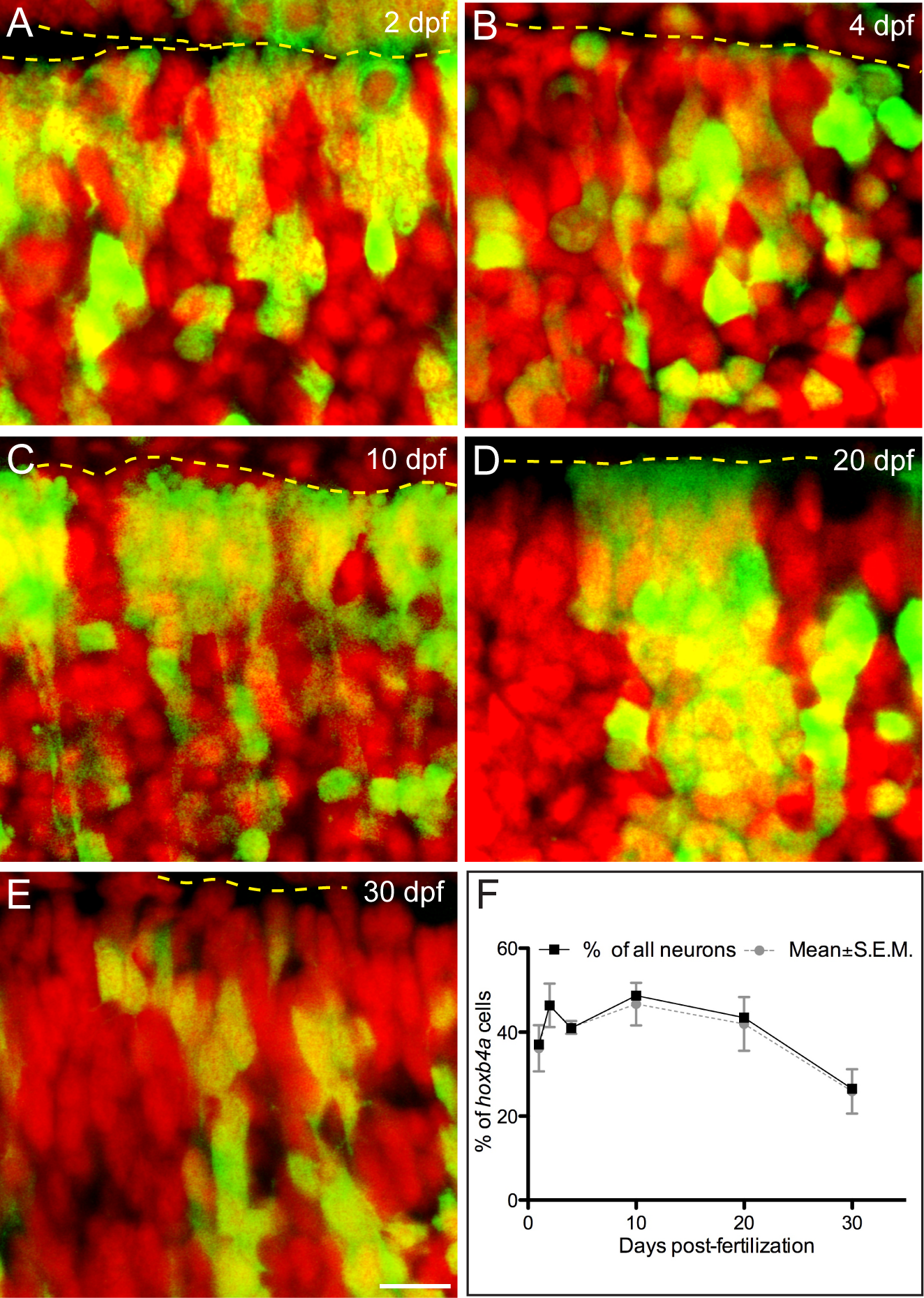

Supplement: Figure S2 — Mosaic hoxb4a activity. (A–D) Single plane images showing hoxb4a (green) and Hoechst nuclear counterstain (red) acquired from the dorsal 60 µm of r7–8 at 2 (A), 4 (B), 10 (C) and 20 (D) dpf in transgenic zebrafish. Dashed lines mark the ventricular surface. (E) Graph showing the percentage change of hoxb4a cells from 1 to 30 dpf in dorsal r7–8. Scale bars = 10 µm. (2.98 MB TIF) [file pone.0005944.s002.tif]

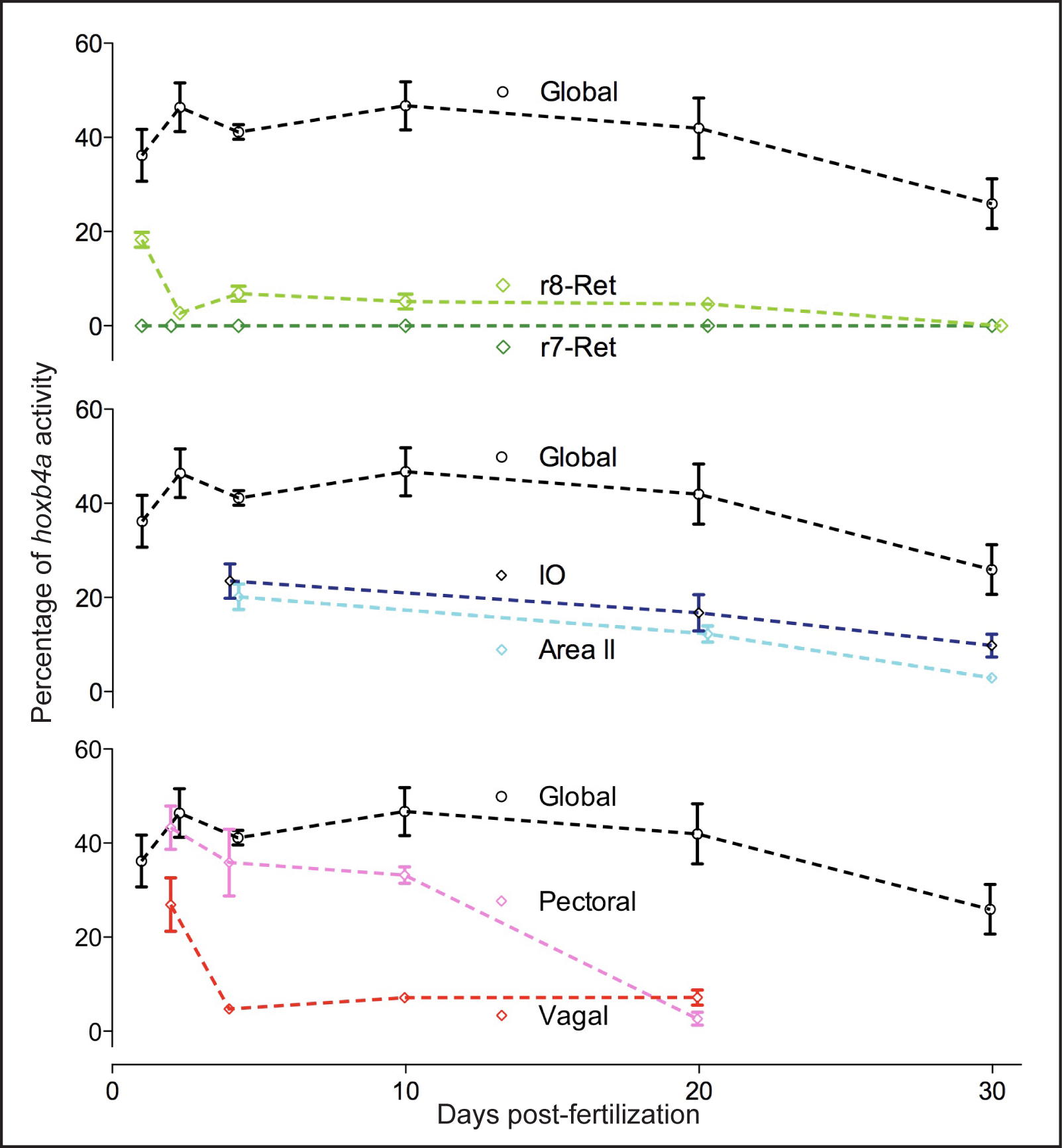

Supplement: Figure S3 — Percentage of hoxb4a cells in each identified neuronal subgroup versus time from 2–30 dpf. Percentages are presented as mean±S.E.M. calculated from individual experiments (also see Table S2) (0.58 MB TIF) [file pone.0005944.s003.tif]

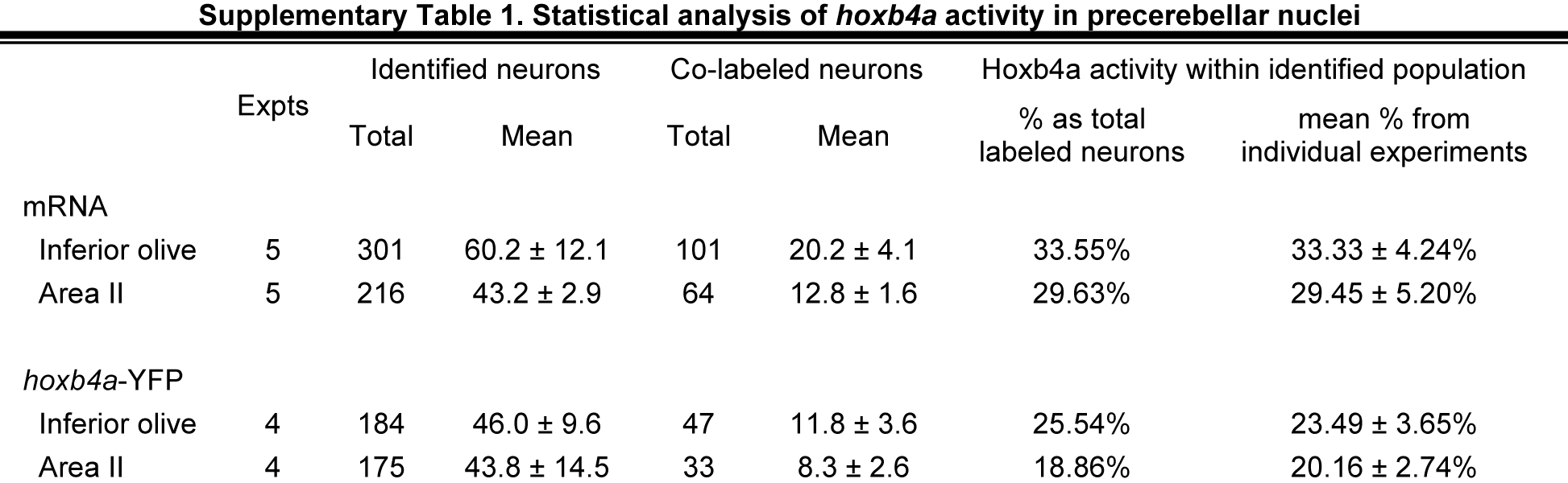

Supplement: Table S1 — Statistical analysis of hoxb4a activity in precerebellar nuclei using mRNA expression and the hoxb4a-YFP reporter. Means are expressed as mean±S.E.M. (0.13 MB TIF) [file pone.0005944.s004.tif]

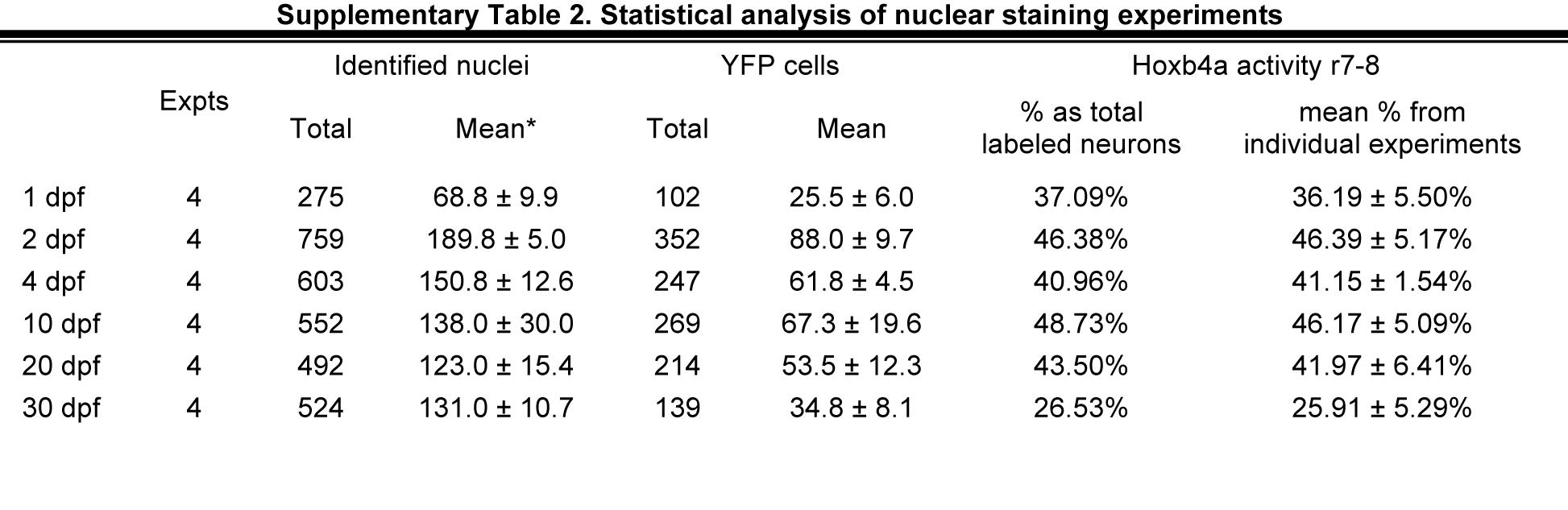

Supplement: Table S2 — Statistical analysis of nuclear counter-stain experiments. Means are expressed as mean±S.E.M. (0.14 MB TIF) [file pone.0005944.s005.tif]

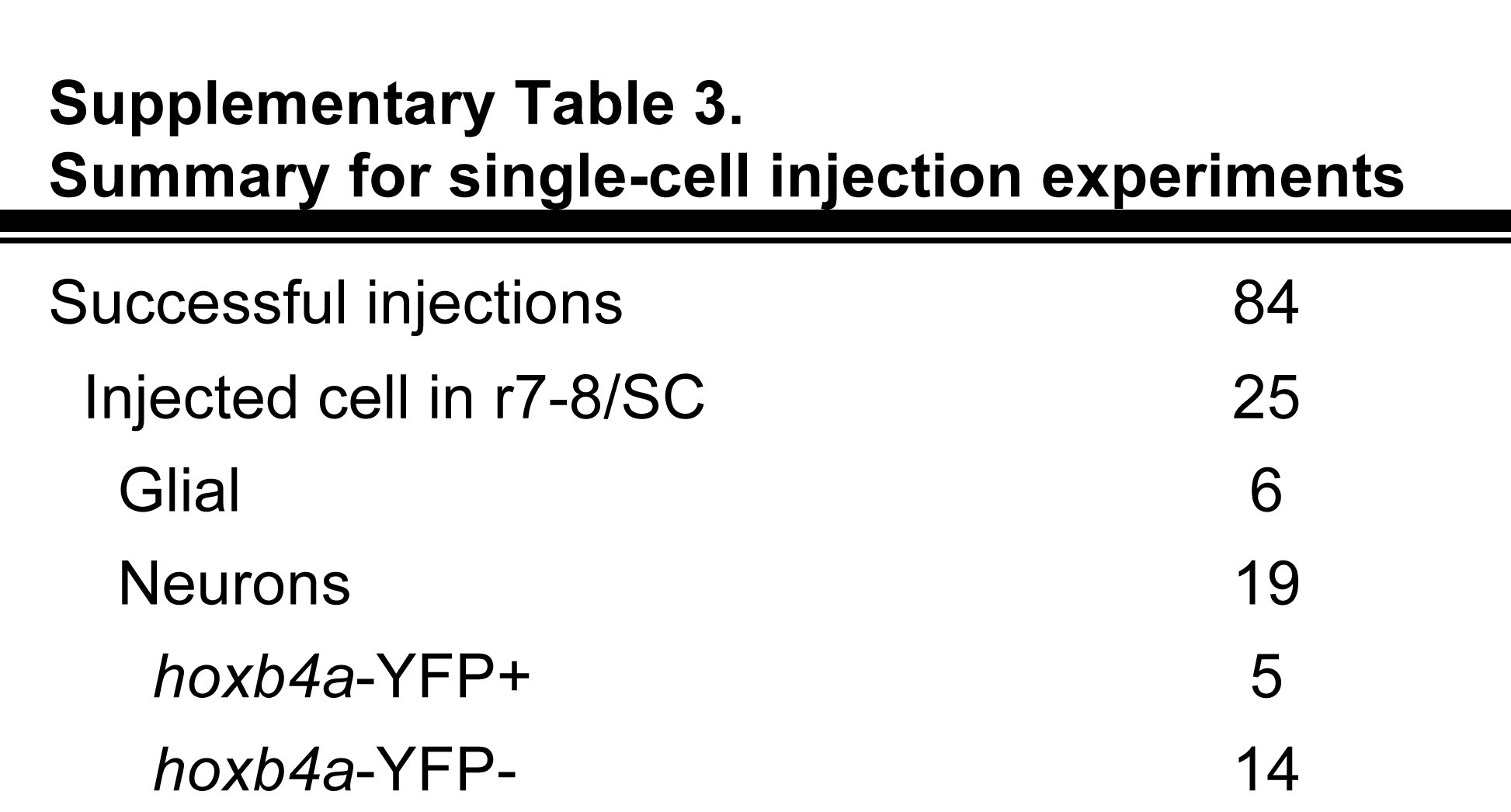

Supplement: Table S3 — Summary of single-cell injection experiments. (0.13 MB TIF) [file pone.0005944.s006.tif]

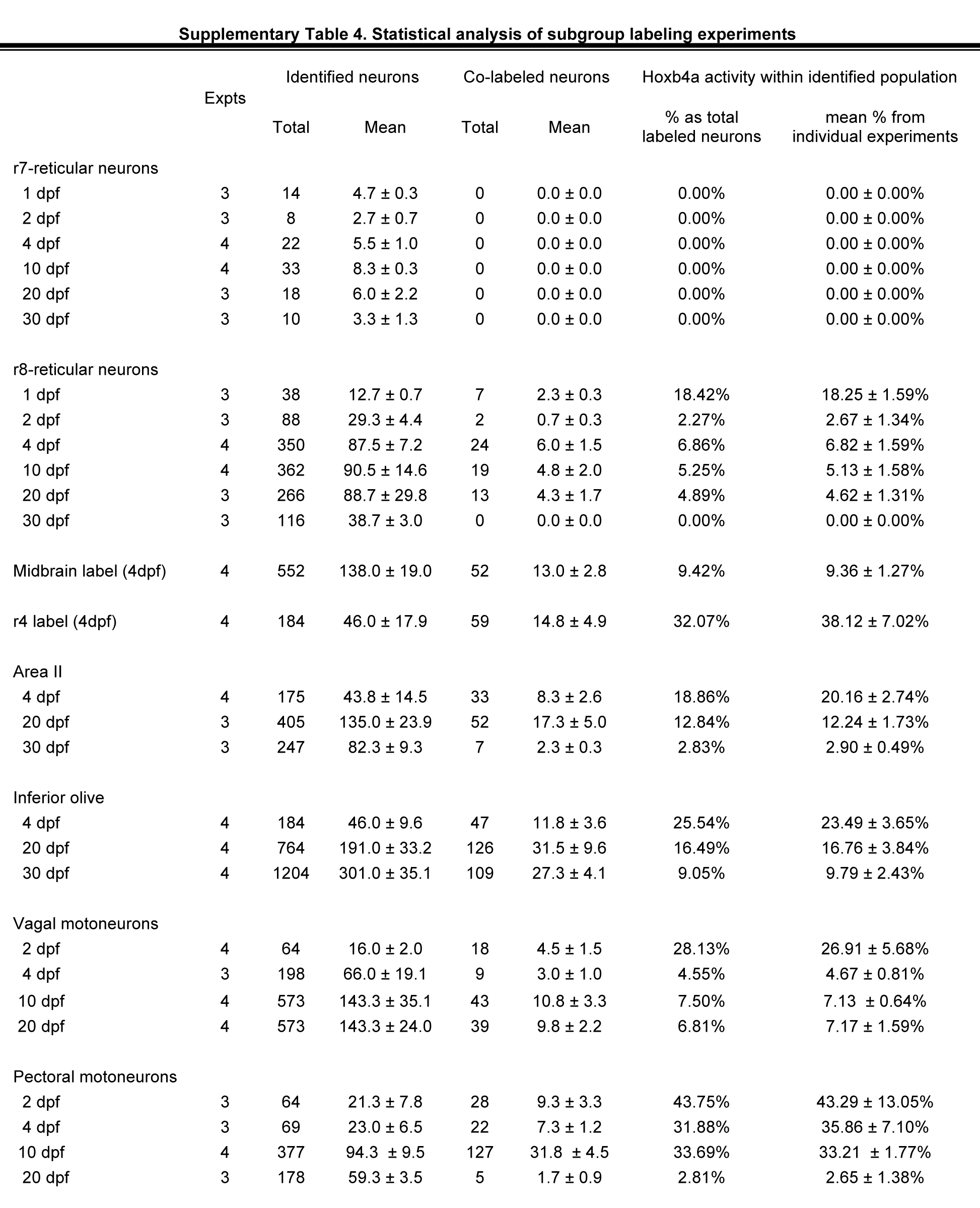

Supplement: Table S4 — Statistical analysis of neuronal subgroup labeling experiments.Numbers of labeled neurons and percentages are expressed as mean±S.E.M. Reticulospinal neurons were not morphologically distinguishable at 1 dpf. The two rostral-most groups of labeled neurons within the hoxb4a-YFP domain were considered to be progenitors that eventually give rise to r7-reticular neurons. (0.38 MB TIF) [file pone.0005944.s007.tif]
